# AI-Driven Quantitative Coronary CT Angiography in Suspected Coronary Artery Disease: Multicenter CONFIRM2 Registry

**DOI:** 10.1016/j.jacadv.2026.102618

**Published:** 2026-03-25

**Authors:** Alexander van Rosendael, Rine Nakanishi, Jeroen J. Bax, Gianluca Pontone, Saima Mushtaq, Ronny R. Buechel, Christoph Gräni, Gudrun Feuchtner, Pietro G. Lacaita, Amit R. Patel, Cristiane C. Singulane, Andrew D. Choi, Mouaz Al-Mallah, Daniele Andreini, Ronald P. Karlsberg, Geoffrey W. Cho, Carlos E. Rochitte, Mirvat Alasnag, Ashraf Hamdan, Filippo Cademartiri, Erica Maffei, Hugo Marques, Pedro de Araújo Gonçalves, Himanshu Gupta, Martin Hadamitzky, Omar Khalique, Dinesh Kalra, James D. Mills, Nick S. Nurmohamed, Paul Knaapen, Matthew Budoff, Kashif Shaikh, Enrico Martin, David M. German, Maros Ferencik, Andrew C. Oehler, Roderick Deaño, Prashant Nagpal, Marly van Assen, Carlo N. De Cecco, Vasileios Kamperidis, Borek Foldyna, Jan M. Brendel, Victor Y. Cheng, Kelley R. Branch, Marcio Bittencourt, Sabha Bhatti, Venkateshwar Polsani, George Wesbey, Rhanderson Cardoso, Ron Blankstein, Augustin Delago, Amit Pursnani, Amro Alsaid, Stephen Bloom, Melissa Aquino, Ibrahim Danad

**Affiliations:** a Department of Cardiology, Leiden University Medical Center, Leiden, the Netherlands; b Department of Cardiovascular Medicine, Toho University Graduate School of Medicine, Tokyo, Japan; c Department of Perioperative Cardiology and Cardiovascular Imaging, Centro Cardiologico Monzino IRCCS, Milan, Italy; d Perioperative Cardiology and Cardiovascular Imaging Department, Centro Cardiologico Monzino IRCCS, Milan, Italy; e Department of Nuclear Medicine, Cardiac Imaging, University Hospital and University of Zurich, Zurich, Switzerland; f Department of Cardiology, Inselspital, Bern University Hospital, University of Bern, Bern, Switzerland; g Department of Radiology, Medical University of Innsbruck, Innsbruck, Austria; h Division of Cardiovascular Medicine, University of Virginia, Charlottesville, Virginia, USA; i Department of Cardiology and Radiology, George Washington University, Washington, DC, USA; j Department of Cardiology, Houston Methodist, Houston, Texas, USA; k Division of University Cardiology, IRCCS Galeazzi Sant’Ambrogio, Department of Biomedical and Clinical Sciences, University of Milan, Italy; l Cardiovascular Research Foundation of Southern California, California, USA; m David Geffen School of Medicine at the University of California-Los Angeles, Los Angeles, California, USA; n Smidt Heart Institute, Cedars-Sinai Medical Center, Los Angeles, California, USA; o Heart Institute, InCor, University of Sao Paulo Medical School, Sao Paulo, Brazil; p Cardiac Center, King Fahad Armed Forces Hospital, Jeddah, Saudi Arabia; q Department of Cardiology, Rabin Medical Center, Petach Tikva, Tel Aviv, Israel; r Gray Faculty of Medical and Health Sciences, Tel Aviv University, Tel Aviv, Israel; s Department of Radiology, IRCCS SYNLAB SDN, Naples, Italy; t UNICA, Unit of Cardiovascular Imaging, Hospital da Luz, Imaging Department, Católica Medical School, Lisbon, Portugal; u CHRC-Comprehensive Health Research Center, LA-REAL, NOVA Medical School, Lisbon, Portugal; v Cardiac Imaging, Heart and Vascular Institute, Valley Health System, Paramus, New Jersey, USA; w Department of Radiology and Nuclear Medicine, German Heart Center Munich, Munich, Germany; x Division of Cardiovascular Imaging, St. Francis Hospital and Heart Center, Roslyn, New York, USA; y Division of Cardiology, Department of Medicine, University of Louisville School of Medicine, Louisville, Kentucky, USA; z Department of Cardiology, West Virginia University Heart & Vascular Institute, Morgantown, West Virginia, USA; aa Department of Cardiology, Amsterdam UMC, Vrije Universiteit Amsterdam, Amsterdam, the Netherlands; bb Department of Vascular Medicine, Amsterdam UMC, University of Amsterdam, Amsterdam, the Netherlands; cc The Lundquist Institute, Torrance, California, USA; dd University of Tennessee Medical Center, Knoxville, Tennessee, USA; ee Division of Cardiology, MercyOne-Iowa Heart Center, Des Moines, Iowa, USA; ff Knight Cardiovascular Institute, Oregon Health & Science University, Portland, Oregon, USA; gg Allegheny Health Network Cardiovascular Institute, Allegheny Health Network, Pittsburgh, Pennsylvania, United States of America; hh Department of Medicine, Division of Cardiovascular Medicine, University of Wisconsin School of Medicine and Public Health, Madison, Wisconsin, USA; ii Department of Radiology, University of Wisconsin School of Medicine and Public Health, Madison, Wisconsin, USA; jj Department of Radiology and Imaging Sciences, Emory University, Atlanta, Georgia, USA; kk 1st Cardiology Department, Medical School, Aristotle University of Thessaloniki, Thessaloniki, Greece; ll Department of Radiology, Cardiovascular Imaging and Research Center, Massachusetts General Hospital and Harvard Medical School, Boston, Massachusetts, USA; mm Minneapolis Heart Institute, Minneapolis, Minnesota, USA; nn Division of Cardiology, Cardiovascular Clinical Trials, University of Washington, Seattle, Washington, USA; oo Heart and Vascular Institute, University of Pittsburgh Medical Center, Pittsburgh, Pennsylvania, USA; pp National institute of Cardiovascular Diseases, Karachi, Pakistan; qq Piedmont Heart Institute, Atlanta, Georgia, USA; rr Department of Cardiovascular Computed Tomography, Scripps Clinic, La Jolla, California, USA; ss Division of Cardiovascular Medicine, Brigham and Women’s Hospital, Boston, Massachusetts, USA; tt Medical Data Research Collaborative, London, England, United Kingdom; uu Department of Medicine, Mount Auburn Hospital, Harvard Medical School, Cambridge, Massachusetts, USA; vv Cardiology, Endeavor NorthShore Cardiovascular Institute, Evanston, Illinois, USA; ww University of Chicago, Pritzker School of Medicine, Chicago, Illinois, USA; xx Department of Cardiac Imaging, Baylor Scott and White, The Heart Hospital Plano, Plano, Texas, USA; yy Midwest Heart & Vascular Associates, Kansas City, Missouri, United States of America; zz Cleerly, Inc., Denver, Colorado, USA; aaa Department of Cardiology, Radboud University Medical Center, Nijmegen, the Netherlands

**Keywords:** artificial intelligence, coronary artery disease, coronary CT, noncalcified plaque volume, plaque quantification, quantitative coronary CT, risk stratification, total plaque volume

## Abstract

**Background:**

Plaque assessment by quantitative coronary CT angiography has demonstrated to correlate highly with intravascular ultrasound and optical coherence tomography, and these modalities have shown strong prognostic value.

**Objectives:**

The purpose of this study was to identify the prognostic value of artificial intelligence–guided quantitative CCTA (AI-QCT) for major adverse cardiovascular events (MACE) against the risk factor–weighted clinical likelihood model.

**Methods:**

The CONFIRM2 (COroNary CT Angiography Evaluation For Evaluation of Clinical Outcomes: An InteRnational, Multicenter Registry) is a multicenter, international, observational cohort study that included patients with clinically indicated CCTA and follow-up for MACE. Patients without cardiac symptoms and prior coronary artery disease (CAD) were excluded. Across the entire coronary artery tree, the presence, extent, and composition of CAD were analyzed by an AI-QCT software, and 24 variables at a patient, vessel, and plaque level were derived, including percent luminal narrowing, remodeling index, plaque volumes (total, calcified, noncalcified, low attenuation), and plaque composition. The primary MACE endpoint was defined as a composite of all-cause death, myocardial infarction (MI), stroke, congestive heart failure, late revascularizations, and hospitalization for unstable angina. The secondary MACE endpoint was defined as all-cause death and MI.

**Results:**

A total of 3,551 patients (age 58.8 ± 12.5 years, 50.5% male) were followed for a median of 4.27 (IQR: 3.47-5.08) years during which 167 (4.7%) events occurred. After excluding collinear variables, diameter stenosis (HR: 1.25 [95% CI: 1.18-1.32]) per 10% increase and noncalcified plaque volume (HR: 1.07 [95% CI: 1.03-1.11]) per 50 mm^3^ increase were the only independent predictors for MACE. In multivariable modeling, the discriminatory value defined by area under the curve (AUC) improved from 0.63 (95% CI: 0.58-0.67) based on the risk factor–weighted clinical likelihood model to 0.76 (95% CI: 0.77-0.80), *P* < 0.001 when adding AI-QCT-based diameter stenosis and noncalcified plaque volume. A similar improvement in risk prediction was seen when adding AI-QCT (AUC 0.77; *P* < 0.001) to a model with traditional risk factors, age, and sex (AUC: 0.67). In addition, AI-QCT significantly improved discrimination compared to the atherosclerotic cardiovascular disease risk score (AUC: 0.63; 95% CI: 0.58-0.68) to 0.75 (95% CI: 0.69-0.80; *P* < 0.001). Similar results were seen for the secondary MACE endpoint of death/MI.

**Conclusions:**

This first multicenter global registry with AI-guided quantitative CT identified noncalcified plaque burden and increment in stenosis severity as the most powerful predictors of MACE, demonstrating the interplay between traditional and novel measures of the severity of CAD. Standardized and rapid quantitative assessment of CAD may improve clinical implementation of multidimensional assessment of CAD as a cornerstone for risk assessment.

Coronary artery disease (CAD) remains a predominant cause of mortality, underscoring the critical need for enhanced identification of patients predisposed to adverse cardiovascular events.^1^ Although numerous risk scores have been developed to refine risk stratification, they primarily extrapolate from traditional cardiovascular risk factors, serving only as a proxy for underlying coronary atherosclerotic burden.^2^ Consequently, these scores lack granularity in prognostication, as they do not directly capture the primary disease process, namely coronary atherosclerosis itself.^3^, ^4^, ^5^, ^6^

Coronary intravascular ultrasound (IVUS) studies have shown that the atheroma burden is one of the strongest predictors for coronary events.^7^ In addition, lesion-specific parameters such as small lumen area or high lipid content associate with lesion-specific risk.^8^^,^^9^ By optical coherence tomography (OCT), the identification of thin cap fibroatheroma and a minimal lumen area <3.5 mm^2^ increased coronary events by a factor 5.^10^ Coronary computed tomography angiography (CCTA) provides a noninvasive approach to characterize the totality of CAD from the entire coronary tree. The emergence of artificial intelligence (AI)–driven methodologies has facilitated accurate, reproducible, standardized, and rapid quantification of coronary atherosclerotic burden and morphology.^11^, ^12^, ^13^ Therefore, quantification of atherosclerosis by CT has become available for clinical practice. Atheroma volume and stenosis degree from CT have shown a close correlation with IVUS.^11^^,^^12^ CT diagnosed low-attenuation plaque (LAP) has shown to correlate strongly with OCT-defined thin cap fibroatheroma.^14^

Limited multicenter studies on the prognostic value of quantitative CT are available. A recent study by Nurmohamed et al demonstrated that total atherosclerotic burden significantly enhances prognostication compared to the mere qualitative CAD-RADS 2.0 in predicting future cardiovascular events.^15^ Specifically, the noncalcified plaque (NCP) content emerges as a key prognostic metric, as NCPs are typically more metabolically active than heavily calcified plaques (CPs), reflecting an unstable plaque phenotype.^16^^,^^17^ Data from the ICONIC (Incident Coronary Syndromes Identified by Computed Tomography) study revealed that densely CPs with ≥1,000 HU, the so-called “1K plaques” are associated with stable disease and a lower risk of adverse events.^17^ In addition, NCP burden represents a modifiable component of total plaque burden and may act as a potential therapeutic target for future interventions.^18^^,^^19^ Nevertheless, the abundance of data generated by quantitative CCTA has not yet been fully leveraged, and the specific quantitative atherosclerotic parameters most predictive of major adverse cardiovascular events (MACE) have yet to be clearly identified.

Therefore, this study aims to evaluate the prognostic value of AI-guided quantitative CCTA (AI-QCT). The discriminative accuracy of AI-QCT-derived atherosclerotic metrics will be compared to traditional risk factors and the risk factor–weighted clinical likelihood (RF-CL) model, and the atherosclerotic cardiovascular disease (ASCVD) score in a large cohort of symptomatic patients suspected of CAD.

## Methods

### Study design and patient population

The CONFIRM 2 (COroNary CT Angiography Evaluation For Evaluation of Clinical Outcomes: An InteRnational, Multicenter Registry) is an ongoing multicenter, international, observational cohort study. Detailed descriptions of the study design have been published previously.^20^ The primary aim of the CONFIRM2 registry is to assess the prognostic value of AI-based quantitative measures of coronary atherosclerosis including plaque burden, morphology, and composition as well as the degree of luminal obstruction for predicting MACE in patients referred for CCTA. Participating sites prospectively and/or retrospectively included sequential patients with a clinically indicated CCTA of ≥64-detector rows. Exclusion criteria for enrollment were absence of CCTA data or follow-up information for clinical events, pregnancy, or a noncardiac illness with life expectancy <2 years. For this analysis, we included patients with cardiac symptoms and without prior CAD (defined as a prior myocardial infarction (MI), prior percutaneous coronary intervention or coronary artery bypass grafting or known ≥50% stenosis on invasive coronary angiography) and who had at least 3 years of follow-up for clinical events, or an event within 3 years. This study was conducted in accordance with the Declaration of Helsinki. Institutional Review Board approval was obtained at each participating center.

### Clinical data collection

Cardiovascular risk factors were systematically collected from the electronic medical record baseline prior to CCTA. Hypertension was defined as a blood pressure of ≥140/90 mm Hg, a documented history of hypertension, or current treatment with antihypertensive medications. Diabetes mellitus was defined by a prior physician diagnosis and/or the use of insulin or oral hypoglycemic agents. Dyslipidemia was defined as either untreated dyslipidemia or current treatment with lipid-lowering medications. Smoking history was classified as positive if the patient was a current smoker or had ceased smoking within 3 months of testing. Family history of CAD was based on patient self-report. Symptom presentation was categorized as typical angina, atypical angina, noncardiac chest pain, dyspnea, palpitations, syncope, other, or asymptomatic. The RF-CL score was then calculated using age, sex, symptoms, and the number of traditional risk factors, as defined in the European Society of Cardiology (ESC) guidelines for the management of chronic coronary syndromes.^21^

### Image acquisition and analysis

CCTA scans were performed using various CT platforms, all of which met the requirement of employing multislice CT scanners with ≥64 slices. The imaging protocol adhered to the guidelines set forth by the Society of Cardiovascular Computed Tomography.^22^ Patient preparation, data acquisition, and image analysis were conducted according to the institutional policies of the participating sites.

Quantitative coronary atherosclerosis evaluation was performed for every coronary artery and its branches using an automated U.S. Food and Drug Administration–cleared AI-enabled software platform (Cleerly Labs, Cleerly Inc). The AI software employs validated convolutional neural networks for image quality assessment, coronary segmentation and labeling, lumen wall evaluation, vessel contour determination, and plaque characterization. Prior validation of the software has been documented in multicenter trials comparing it to expert consensus, quantitative coronary angiography, fractional flow reserve, and IVUS.^11^^,^^13^ This analysis was performed on all CCTA scans and only coronary segments with a diameter of ≥1.5 mm were included. Each segment was assessed for the presence of coronary atherosclerosis, defined as any tissue structure ≥1 mm^3^ within the coronary artery wall that could be distinguished from surrounding epicardial tissue, epicardial fat, or the vessel lumen. Plaque volumes (in mm^3^) were calculated for each coronary lesion and summed to determine the total plaque volume (TPV) at the patient level. Plaque types are categorized based on HU ranges: LAP as <30 HU, NCP as HU between 30 and 350, and CP as >350 HU.^23^ High-risk plaque (HRP) was defined as a lesion with LAP ≥2 mm^3^ and a remodeling index of >1.1. This definition is based on Omori et al,^11^ since this magnitude of LAP and vessel remodeling correlated most strongly with near-infrared spectroscopy-IVUS detected lipid core. Plaque burden normalized to the vessel volume was also reported as percent atheroma volume (PAV), calculated as (plaque volume/vessel volume) × 100%. The vessel volume includes all coronary segments with a diameter ≥1.5 mm, regardless of plaque presence, differing from other quantitative CT software that may include only segments containing plaque. The quantification software detects small amount of plaque which results in a low prevalence of completely normal CCTA and high prevalence of nonobstructive stenosis. The quantification process captures very small plaques that are not visually identifiable and that would previously have been considered normal. To circumvent this issue, a threshold of 16 mm^3^—corresponding to the lowest quintile of nonobstructive CAD—was applied to define significant plaque. In cases where image quality was degraded by motion, insufficient opacification, beam hardening, or other artifacts, only the portions of the coronary artery with compromised quality were omitted from the analysis. Finally, a credentialed and trained radiologic technologist provides quality assurance overview of the AI analysis.

### Endpoints

The primary endpoint was the incidence rate of MACE over a minimum follow-up of 3 years. MACE was defined as a composite of all-cause mortality, MI, stroke, congestive heart failure, late revascularizations (occurring >90 days post index CCTA), and hospitalization for unstable angina. Death status was confirmed through signed verification forms submitted by the local principal investigators. The secondary endpoint was a composite of all-cause mortality and MI. MACE was recorded and adjudicated by a Clinical Events Committee.

### Clinical endpoint definitions

#### *Death* from any cause

MI will be defined in accordance to the Universal Definition of MI as established by the European Society of Cardiology, American College of Cardiology (ACC), American Heart Association (AHA), and World Heart Federation.

*Unstable angina-related hospitalization* will be defined as hospitalization for signs or symptoms of unstable angina. Unstable angina will be defined by: 1) rest angina; 2) new-onset angina (<2 months); or 3) increasing angina (in intensity, duration, and/or frequency. Patients hospitalized for unstable angina will be considered as having experienced CAD-related hospitalization, whether target vessel revascularization is performed or not.

*Late coronary revascularization*, which will be defined as revascularization occurring ≥90 days following the index diagnostic test.

*Cerebrovascular accident* will be defined as a neurological deficit lasting ≥24 hours or lasting <24 hours with a brain imaging study showing infarction.

*Congestive heart failure* was defined according to the Framingham Heart Failure Diagnostic Criteria, requiring either two major criteria or one major and two minor criteria to be present concurrently.^24^

### Statistical analysis

Descriptive data are presented as median and IQR for nonparametric data and as mean ± SD for parametric data. Categorical variables are expressed as absolute numbers and percentages. Cox proportional hazards regression was used for outcome analyses. Univariable analyses included 24 candidate CT metrics based on clinical utility and prior studies. The following CT metrics were evaluated for their prognostic value in the present analysis: percent diameter stenosis, percent area stenosis, number of moderate stenoses (50% to 69%), number of severe stenoses (≥70%), TPV, CP volume, NCP volume, LAP volume, percent atheroma volume (PAV), PAV CP, PAV NCP, PAV LAP, number of HRPs, plaque diffuseness (TPV/vessel length), total vessel length, left main with moderate stenosis, left main with severe stenosis, proximal left anterior descending artery (LAD) with moderate stenosis, proximal LAD with severe stenosis, number of chronic total occlusions (100% stenosis), lumen volume, vessel volume, lumen volume/vessel length, and minimal luminal diameter. Definitions are provided in Supplemental Table 1. A logistic regression model was employed to exclude collinear variables (variance inflation factor >5). Using backward selection (*P* < 0.05), a parsimonious multivariable model was created to identify independent predictors of MACE among candidate AI-QCT variables. The predictive performance of the multivariable model was assessed using the area under the receiver-operating characteristic curve (AUC). In addition, the Harrel’s C-statistic was calculated to account for censored time-to-events for prediction of outcome. After that, we focused on a stepwise approach simulating clinical decision-making. We chose the RF-CL risk model as our base model. In addition, a model was created with only traditional cardiovascular risk factors, age and sex to evaluate the incremental prognostic information of AI-QCT to this model. In a different approach geared toward cardiovascular risk assessment, we chose the ASCVD risk score as a base model and then added the AI-QCT measures in the second model. Cumulative incidence curves were generated for tertiles of risk derived from the AI-enabled quantitative CCTA model. Kaplan-Meier survival analyses were performed to estimate event rates across RF-CL risk categories. Additional stratification was conducted according to the median NCP volume. All statistical analyses were performed using SAS 9.4 (SAS Institute Inc). A *P* value <0.05 was considered as statistically significant.

## Results

This analysis included 4,163 symptomatic patients from 18 centers across 11 countries, recruited between 2008 and 2022. After excluding 288 asymptomatic patients, 322 patients with a prior cardiac history, and 2 patients with incomplete clinical information, the final cohort comprised 3,551 patients. The mean age of the cohort was 58.8 ± 12.5 years, with 50.5% male patients and 80.2% of Caucasian ethnicity (Table 1). Symptom presentation included typical angina in 19.1%, noncardiac chest pain in 54.2%, and other symptoms in the remaining patients. The risk calculated by the RF-CL model was very low, low, and moderate in 30%, 35%, and 20.9% of patients, respectively. Information to calculate the ASCVD risk score was only available in 1,309 (37%) patients. Approximately two-thirds of the cohort were on statin therapy at baseline (Supplemental Table 2). Over a median follow-up period of 4.27 (IQR: 3.47-5.08), 167 (5%) patients experienced a MACE: 34 (20%) died, 24 (14%) had a nonfatal MI, 12 (7%) had a nonfatal stroke, 23 (14%) patients were hospitalized for chronic heart failure, 17 (10%) patients were hospitalized for unstable angina, and 84 (50%) underwent a late revascularization (62 percutaneous coronary intervention, 17 coronary artery bypass graft, and 5 unspecified). Patients who experienced events were older, predominantly male, and had a higher prevalence of cardiovascular risk factors (Table 1).

**Table 1:** Baseline Demographics and Risk Factors

	All Patients (N = 3,551)	Patients Without Events (n = 3,384, 95.0%)	Patients With Events (n = 167, 5.0%)	*P* Value
Age (y)	58.81 ± 12.45	58.64 ± 12.49	62.18 ± 11.06	0.0006
Male	1792 (50.5%)	1,682 (49.7%)	110 (65.9%)	<0.0001
Race				0.4779
Asian	123 (3.5%)	116 (3.4%)	7 (4.2%)	
Black or African American	174 (4.9%)	167 (4.9%)	7 (4.2%)	
White	2,809 (79.1%)	2,671 (78.9%)	138 (82.6%)	
Other	445 (12.5%)	430 (12.7%)	15 (9.0%)	
Ethnicity				0.0016
Hispanic or Latino	319 (9.0%)	314 (9.3%)	5 (3.0%)	
Not Hispanic or Latino	2,148 (60.5%)	2024 (59.8%)	124 (74.3%)	
Missing	1,084 (30.5%)	1,046 (30.9%)	38 (22.8%)	
Cardiovascular risk factors				
Body mass index, kg/m^2^	27.90 ± 5.77	27.87 ± 5.80	28.39 ± 5.18	0.1335
Smoker	498 (14.0%)	463 (13.7%)	35 (21.0%)	0.0100
Diabetes	530 (14.9%)	483 (14.3%)	47 (28.1%)	<0.0001
Hypertension	1929 (54.3%)	1815 (53.6%)	114 (68.3%)	0.0003
Dyslipidemia	1708 (48.1%)	1,614 (47.7%)	94 (56.3%)	0.0381
Family history positive for CAD	1,014 (28.6%)	965 (28.5%)	49 (29.3%)	0.8178
Atrial fibrillation	283 (8.0%)	259 (7.7%)	24 (14.4%)	0.0045
Heart failure	199 (5.6%)	173 (5.1%)	26 (15.6%)	<0.0001
Peripheral artery disease	64 (1.8%)	60 (1.8%)	4 (2.4%)	0.5473
Cardiac symptoms				
Typical angina	678 (19.1%)	634 (18.7%)	44 (26.3%)	0.0146
Atypical angina	1,335 (37.6%)	1,277 (37.7%)	58 (34.7%)	0.4337
Noncardiac chest pain	590 (16.6%)	563 (16.6%)	27 (16.2%)	0.8736
Dyspnea	753 (21.2%)	722 (21.3%)	31 (18.6%)	0.3921
Palpitations	202 (5.7%)	194 (5.7%)	8 (4.8%)	0.6077
Syncope	67 (1.9%)	62 (1.8%)	5 (3.0%)	0.2439
Other symptoms	575 (16.2%)	547 (16.2%)	28 (16.8%)	0.8366
ASCVD score				0.0002
>0-5	411 (11.6%)	402 (11.9%)	9 (5.4%)	
>5-7.5	161 (4.5%)	151 (4.5%)	10 (6.0%)	
>7.5-20	450 (12.7%)	412 (12.2%)	38 (22.8%)	
>20	287 (8.1%)	260 (7.7%)	27 (16.2%)	
Not available	2,242 (63.1%)	2,159 (63.8%)	83 (49.7%)	
Risk factor–weighted clinical likelihood (RF-CL)				<0.0001
Very low	1,067 (30.0%)	1,040 (30.7%)	27 (16.2%)	
Low	1,243 (35.0%)	1,184 (35.0%)	59 (35.3%)	
Moderate	742 (20.9%)	681 (20.1%)	61 (36.5%)	
Missing	499 (14.1%)	479 (14.2%)	20 (12.0%)	

ASCVD = atherosclerotic cardiovascular disease; CAD = coronary artery disease.

### AI-QCT atherosclerotic measurements

The prevalence of CAD in this cohort was 93.0% with all of those patients demonstrating NCP, and 2,577 (72.6%) showing CP. The median TPV per patient was 56.8 mm^3^ (IQR: 16.0, 187.6), with NCP accounting for the majority (41.90 mm^3^; IQR: 12.90, 114.80), Table 2. The median CP volume was 6.6 mm^3^ (IQR: 0.0, 54.2). A histogram of NCP volume (Supplemental Figure 1) revealed that the largest subgroup had an NCP volume between 15 and 50 mm^3^ (27% of patients). LAP >2 mm^3^ was present in 6.2% of patients, while HRP (LAP ≥ 2 mm^3^ with a remodeling index >1.1) was found in 5.1% of patients. No or minimal CAD, nonobstructive CAD, and 1-, 2-, and 3-vessels or left main obstructive (≥50%) CAD was present in 25.0%, 60.4%, 9.9%, 3.2%, and 1.4% of patients, respectively.

**Table 2:** AI-QCT Atherosclerotic Features Stratified by Patients With and Without Events

	All Patients (N = 3,551)	Patients Without Events (n = 3,384, 95.0%)	Patients With Events (n = 167, 5.0%)	*P* Value
Plaque volumes				
Total plaque volume, mm^3^				<0.0001
Median (IQR)	56.80 (16.00, 87.60)	53.30 (15.05,170.55)	260.70 (84.50, 482.00)	
Mean, SD	152.1 ± 245.1	142.20 ± 233.80	353.13 ± 358.23	
Number of patients with any plaque	3,301 (93.0%)	3,134 (92.6%)	167 (100.0%)	
Noncalcified plaque volume, mm^3^				<0.0001
Median (IQR)	41.90 (12.90,114.80)	39.60 (12.25,107.40)	148.80 (60.30, 296.2)	
Mean, SD	91.47 ± 136.36	85.65 ± 128.12	209.55 ± 220.10	
Number of any noncalcified plaque	3,300 (93.0%)	3,133 (92.6%)	167 (100.0%)	
Calcified plaque volume, mm^3^				<0.0001
Median (IQR)	6.60 (0.00,54.20)	5.90 (0.00,48.45)	53.70 (5.50, 206.90)	
Mean, SD	59.67 ± 134.49	55.63 ± 129.03	141.51 ± 201.17	
Number of patients with any calcified plaque	2,577 (72.6%)	2,428 (71.7%)	149 (89.2%)	
Stenosis				
Diameter stenosis, %				<0.0001^a^
0%	348 (9.8%)	347 (10.3%)	1 (0.6%)	
1%-24%	1896 (53.4%)	1846 (54.6%)	50 (29.9%)	
25%-49%	790 (22.2%)	747 (22.1%)	43 (25.7%)	
50%-69%	283 (8.0%)	249 (7.4%)	34 (20.4%)	
70%-99%	146 (4.1%)	121 (3.6%)	25 (15.0%)	
100%	88 (2.5%)	74 (2.2%)	14 (8.4%)	
Extent of vessel disease				<0.0001^a^
None/minimal (≤16 mm^3^ of plaque volume^b^)	889 (25.0%)	881 (26.0%)	8 (4.8%)	
Nonobstructive (>16 mm^3^ of plaque volume^b^)	2,145 (60.4%)	2059 (60.8%)	86 (51.5%)	
1V	351 (9.9%)	307 (9.1%)	44 (26.3%)	
2V	115 (3.2%)	98 (2.9%)	17 (10.2%)	
3V or LM CAD	51 (1.4%)	39 (1.2%)	12 (7.2%)	
Other				
Percent atheroma volume, median (IQR)	2.17 (0.63,6.66)	2.04 (0.59,6.11)	9.29 (3.45,17.59)	<0.0001^a^
Low attenuation plaques >2 mm^3^ present	221 (6.2%)	193 (5.7%)	28 (16.8%)	<0.0001^a^
Number of plaques with LAP >2 mm^3^				<0.0001^a^
0	3,330 (93.8%)	3,191 (94.3%)	139 (83.2%)	
1	159 (4.5%)	138 (4.1%)	21 (12.6%)	
2	45 (1.3%)	39 (1.2%)	6 (3.6%)	
≥3	17 (0.5%)	16 (0.5%)	1 (0.6%)	
High-risk plaque present	210 (5.9%)	184 (5.4%)	26 (15.6)	<0.0001^a^
Number of high-risk plaques^a^				<0.0001^a^
0	3,341 (94.1%)	3,200 (94.6%)	141 (84.4%)	
1	60 (1.7%)	51 (1.5%)	9 (5.4%)	
2	92 (2.6%)	79 (2.3%)	13 (7.8%)	
3	37 (1.0%)	33 (1.0%)	4 (2.4%)	
>3	21 (0.6%)	21 (0.6%)	0 (0.0%)	

AI-QCT = artificial intelligence–guided quantitative CCTA; LAP = low-attenuation plaque; LM = left main; other abbreviation as in Table 1.

a Defined as LAP ≥ 2 mm^3^ and remodeling index >1.1.

b 16 mm^3^ plaque volume is the lowest quintile of nonobstructive CAD.

### Prognostic value of CT-derived atherosclerotic characteristics in risk stratification

**Figure 1:**
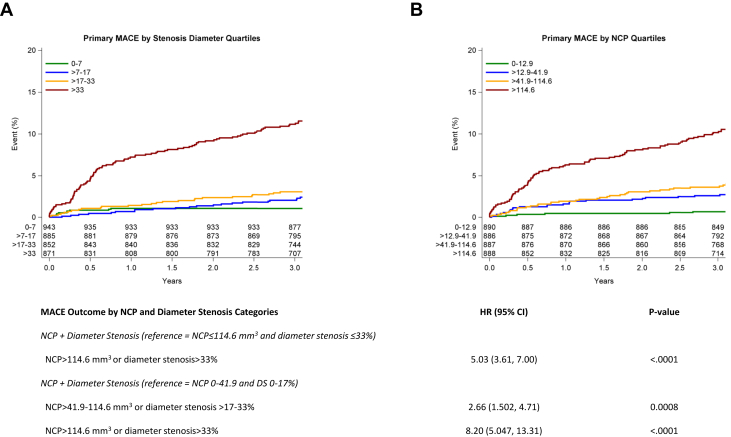
Event Rates Across Stenosis and Noncalcified Plaque Volume Quartiles MACE prediction by quartiles of diameter stenosis (A) and noncalcified plaque volume (B). MACE = major adverse cardiovascular events; NCP = noncalcified plaque volume.

Patients with events had significantly higher plaque volumes than those without an event with TPV, NCP volume, and CP volume being approximately 5-, 3.5-, and 10-fold greater, respectively (Table 2). Obstructive CAD was present in 43.8% of patients with events, whereas only 4.8% of these patients with events had no or minimal CAD. Additionally, patients who experienced a MACE had a significantly higher number of HRPs. Among 24 univariable candidate variables, all except average lumen area and severe left main stenosis (due to the very low prevalence, n = 1) were significantly associated with MACE (Supplemental Table 3). HRs for TPV, NCP volume, and CP volume were 1.07 (95% CI: 1.06-1.09), 1.14 (95% CI: 1.12-1.17), and 1.11 (95% CI: 1.08-1.13), respectively, per 50 mm^3^ of plaque. With increasing tertiles of TPV, NCP, and CP, NCP demonstrated the highest HR per tertile increase compared with the other compositional plaque volumes for the prediction of both the primary (Supplemental Table 4A) and secondary endpoint (Supplemental Table 4B). An increase in NCP tertile resulted in a 2.33 higher risk of death and MI, compared to a relative risk of 2.17 and 2.02 per tertile increase for TPV and CP, respectively. Figure 1 shows the increase in event rates for MACE by quartiles of diameter stenosis and NCP. Patients with an NCP >41.9 to 114.6 mm^3^ or a diameter stenosis >17 to 33% have a 2.66-fold (95% CI: 1.50-4.71) increased risk of developing MACE compared to those with values below this threshold (*P* = 0.0008). Patients in the highest quartile of NCP and diameter stenosis have an HR of 5.03 (95% CI: 3.61-7.00; *P* < 0.001) compared to those with an NCP volume ≤114.6 mm^3^ or a diameter stenosis ≤33% (Figure 1).

### Discriminatory value of traditional risk factors, RF-CL model, ASCVD, and the AI-QCT model during follow-up

**Figure 2:**
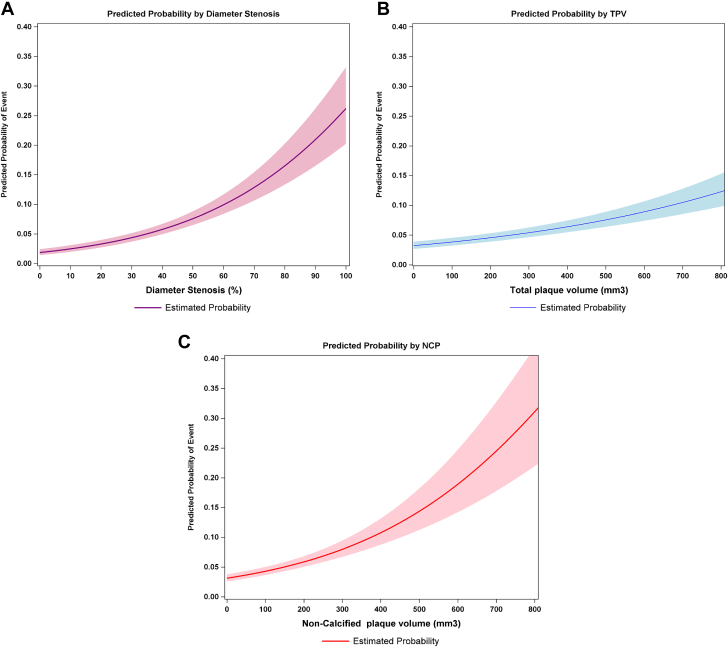
Predicted Probability of Events for the Primary Endpoint Predicted probability of events for the primary endpoint by diameter stenosis (A), TPV (B), and NCP volume (C). TPV = total plaque volume; other abbreviation as in Figure 1.

Diameter stenosis (HR: 1.25 [95% CI: 1.18-1.32]) per 10% increase and NCP volume (HR: 1.07 [95% CI: 1.03-1.11]) per 50 mm^3^ increase were the only independent predictors for MACE 3. In multivariable model with traditional cardiovascular risk factors, age, sex, and the independent univariable predictors of the CT metrics, only diabetes (HR: 1.48 [95% CI: 1.02-2.16]), smoking (HR: 1.66 [95% CI: 1.12-2.45]) diameter stenosis per 10% increase (HR: 1.21 [95% CI: 1.14-1.29]), and NCP volume per 50 mm^3^ increase (HR: 1.07 [95% CI: 1.03-1.11]) remained independent predictors of MACE (Table 3). The addition of NCP and TPV to diameter stenosis significantly improves risk stratification, whereas adding diameter stenosis to a model already containing NCP or TPV has no incremental value beyond these metrics (Table 4). Figure 2 shows the predicted probability of events by diameter stenosis, TPV, and NCP volume. Supplemental Figure 2 shows the predicted probability of events for the secondary endpoint.

**Table 3:** Stepwise Multivariable Modeling for the Primary Outcome

	Multivariable HR (95% CI)	AUC	*P* Value
RF-CL^a^		0.625 (0.582, 0.667)	<0.0001 (*P* value testing the hypothesis that c = 0.5)
Very low	Ref.	--	--
Low	1.89 (1.20, 2.98)		
Moderate	3.33 (2.12, 5.24)		
ASCVD^a^		0.632 (0.580, 0.684)	0.5981 (vs RF-CL)
ASCVD risk score			
0%-5%	Ref.		
5%-7.5%	2.88 (1.17, 7.09)		
7.5%-20%	4.01 (1.94, 8.30)		
>20%	4.48 (2.11, 9.52)		
Optimal AI-QCT model		0.755 (0.719, 0.792)	<0.0001 (vs RF-CL)
Lumen diameter stenosis, per 10%	1.25 (1.18, 1.32)		
Noncalcified plaque volume, per 50 mm^3^	1.07 (1.03, 1.11)		
Risk factors + age + sex + optimal AI-QCT model		0.774 (0.737, 0.810)	<0.0001 (vs RF-CL)
Diabetes	1.48 (1.02, 2.16)	--	--
Hypertension	1.15 (0.81, 1.64)	--	--
Smoking	1.66 (1.12, 2.45)	--	--
Dyslipidemia	0.98 (0.70, 1.37)	--	--
Age (per 1-year increase)	1.01 (0.99, 1.03)	--	--
Sex (male vs female)	1.34 (0.93, 1.95)	--	--
Lumen diameter stenosis, per 10%	1.21 (1.14, 1.29)	--	--
Noncalcified plaque volume, per 50 mm^3^	1.07 (1.03, 1.11)	--	--
ASCVD + optimal AI-QCT model		0.747 (0.693, 0.800)	<0.0001 (vs RF-CL)
ASCVD risk score		--	--
0%-5%	Ref.		
5%-7.5%	2.39 (0.97, 5.89)		
7.5%-20%	2.50 (1.18, 5.30)		
>20%	2.28 (1.03, 5.06)		
Lumen diameter stenosis, per 10%	1.19 (1.09, 1.29)	--	--
Noncalcified plaque volume, per 50 mm^3^	1.07 (1.02, 1.13)	--	--
RF-CL + optimal AI-QCT model		0.762 (0.772, 0.802)	<0.0001 (vs RF-CL)
RF-CL risk score		--	--
Very low	Ref.		
Low	1.41 (0.89, 2.23)		
Moderate	1.61 (0.98, 2.62)		
Lumen diameter stenosis, per 10%	1.22 (1.15, 1.30)	--	--
Noncalcified plaque volume, per 50 mm^3^	1.07 (1.04, 1.11)	--	--
RF-CL + optimal AI-QCT model + statins		0.758 (0.709, 0.807)	<0.0001 (vs RF-CL)
RF-CL risk score		--	--
Very low	Ref.		
Low	1.53 (0.79, 2.96)		
Moderate	1.96 (0.99, 3.86)		
Lumen diameter stenosis, per 10%	1.25 (1.16,1.36)	--	--
Noncalcified plaque volume, per 50 mm^3^	1.03 (0.97, 1.09)	--	--
Statins^a^	1.18 (0.70,1.99)	--	--

RF-CL = risk factor–weighted clinical likelihood model; other abbreviations as in Tables 1 and 2.

a ASCVD score was available in 1,309 patients, statins were available in 2,192 patients, and RF-CL was available in 3,052 patients.

**Table 4:** Evaluation of the Incremental Prognostic Value of Diameter Stenosis Beyond Noncalcified Plaque and Total Plaque Volume

Model	AUC (95% CI)	Estimate Difference (95% CI)	*P* Value
Diameter stenosis alone	0.744 (0.707, 0.781)	0.011 (0.003, 0.019)	DS alone vs DS + NCP = 0.0051
Diameter stenosis + NCP	0.755 (0.719, 0.792)		
NCP alone	0.743 (0.706, 0.781)	0.012 (−0.012, 0.035)	NCP alone vs NCP + DS = 0.3229
NCP + diameter stenosis	0.755 (0.719, 0.792)		
Diameter stenosis alone	0.744 (0.707, 0.781)	0.008 (0.002, 0.014)	DS alone vs DS + TPV = 0.0125
Diameter stenosis + TPV	0.752 (0.715, 0.788)		
TPV alone	0.742 (0.705, 0.780)	0.009 (−0.013, 0.032)	TPV alone vs DS + TPV = 0.4168
Diameter stenosis + TPV	0.752 (0.715, 0.788)		

DS = diameter stenosis; NCP = noncalcified plaque; TPV = total plaque volume.

Receiver-operating characteristic analysis revealed that the addition of AI-QCT to RF-CL model improved the AUC from 0.63 (95% CI: 0.58-0.67) to 0.76 (95% CI: 0.77-0.80; *P* < 0.001) (Central Illustration). When statin use was added to the model of RF-CL and AI-QCT, the performance was similar to the model without correction for statin use (both an AUC of 0.76) (Table 3). The addition of AI-QCT improved risk prediction significantly compared to the model of age, sex, and traditional risk factors (AUC: 0.67 vs 0.77; *P* < 0.001) (Table 3). The ASCVD model showed an AUC of 0.63 (95% CI: 0.58-0.68), which improved to 0.75 (95% CI: 0.69-0.80) with the addition of AI-QCT. For the prediction of the secondary endpoint, similar results were observed, with the AI-QCT model outperforming the RF-CL model, the model including traditional risk factors, and the ASCVD risk score model in predicting all-cause mortality and nonfatal MI) (Supplemental Table 5). The AUC for predicting death and MI for the RF-CL model was 0.57 (95% CI: 0.49-0.65), the addition of the AI-QCT model significantly improved the AUC to 0.73 (95% CI: 0.67-0.81); *P* = 0.002, as shown in Supplemental Table 5). AI-QCT also improved risk stratification compared with the ASCVD score, pertaining to the secondary outcome of death and MI (Supplemental Table 5). Subgroup analyses stratified by sex and age categories consistently demonstrated that the AI-QCT model outperformed traditional risk factors and the RF-CL model in predicting MACE (Supplemental Tables 6 to 9). Even in a subgroup analysis of patients without obstructive CAD (N = 3,034), the discriminatory value of the AI-QCT model was maintained (AUC: 0.70, [95% CI: 0.65-0.75]) (Supplemental Table 10). Supplemental Table 11 presents the Harrell’s C-statistic alongside the AUC values, demonstrating results consistent with the AUC analysis.

**Central Illustration:**
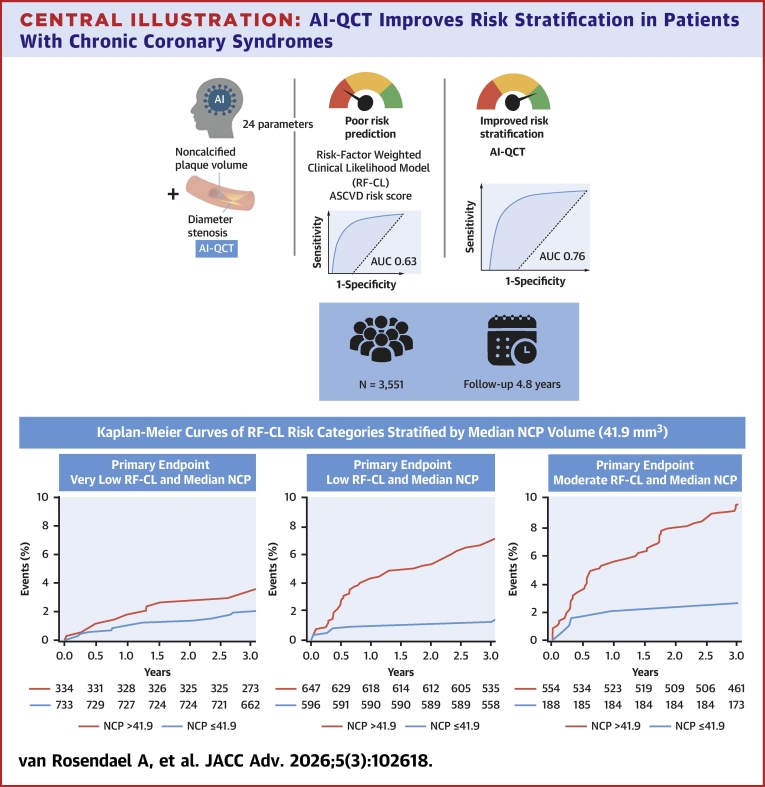
**AI-QCT Improves Risk Stratification in Patients With Chronic Coronary Syndromes** Central illustration showing that an artificial intelligence–based quantitative coronary computed tomography algorithm automatically extracts 24 parameters of coronary atherosclerosis from CCTA. Among these, only diameter stenosis and noncalcified plaque volume (AI-QCT) emerged as independent predictors of MACE. In contrast, the RF-CL model and the ASCVD risk score demonstrated poor discriminative performance for MACE prediction. AI-QCT provided incremental prognostic value beyond both RF-CL and ASCVD scores, enabling improved risk stratification in patients with chronic coronary syndromes. Notably, individuals with a median noncalcified plaque volume >41.9 mm^3^ were at significantly higher risk of MACE, irrespective of their RF-CL risk category. Together, these findings highlight the ability of AI-QCT to refine risk assessment. ASCVD = atherosclerotic cardiovascular disease; CCTA = coronary computed tomography angiography; other abbreviations as in Figures 1 and 3.

### Cumulative incidence curves

**Figure 3:**
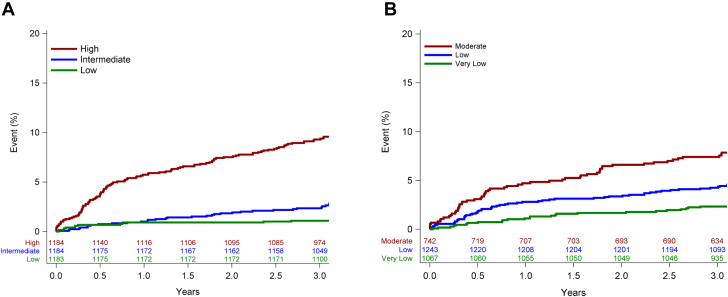
Cumulative Incident Curves for the Primary Endpoint The 3-year event rates are plotted for the AI-QCT model divided by tertiles (A), and for the (B) RF-CL model risk categories. AI-QCT = artificial intelligence quantitative CT; RF-CL = risk factor–weighted clinical likelihood.

**Figure 4:**
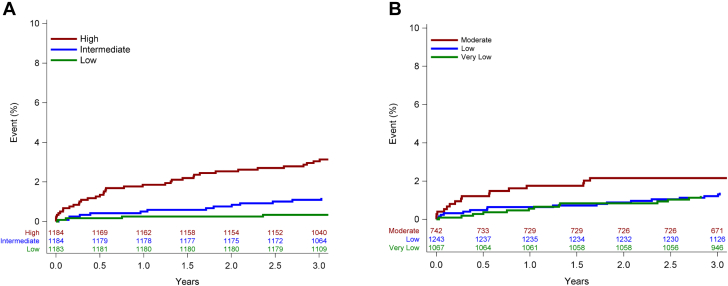
**Cumulative Incident Curves for the Secondary Endpoint** The 3-year event rates are plotted for the AI-QCT model divided by tertiles (A), and for the RF-CL model risk categories (B). Patients with NCP volumes above the median consistently demonstrated higher event rates within each RF-CL risk category compared with those with NCP volumes at or below the median. Numbers at risk for each group are provided below the x-axis. Abbreviations as in Figures 1 and 3.

**Figure 5:**
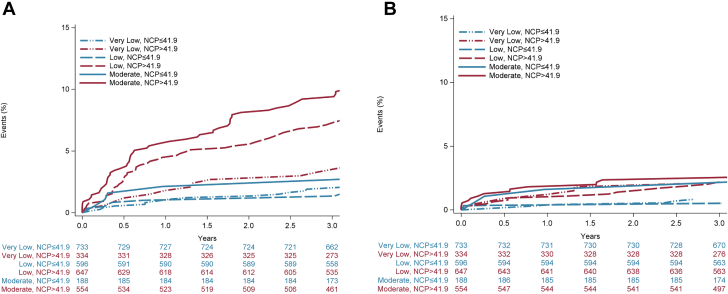
Kaplan-Meier Events by RF-CL Risk and Noncalcified Plaque Volume Kaplan-Meier curves showing event rates across RF-CL risk categories stratified by median NCP volume (cutoff 41.9 mm^3^) for the primary endpoint (A) and secondary endpoint (B). MI = myocardial infarction; other abbreviations as in Figures 1 and 3.

The 3-year event rates for patients classified as low, intermediate, and high risk based on the optimal AI-QCT model were 1.3%, 3.2%, and 9.9%, respectively (Figure 3). The median NCP volumes for these groups were 8.0 mm^3^, 42.6 mm^3^, and 160.0 mm^3^, respectively, with mean stenosis values of 4.3%, 17.7%, and 49.9%, respectively. In comparison, the event rates for the very low, low, and moderate RF-CL calculated risk were 2.6%, 4.8%, and 8.3%, respectively. Notably, the AI-QCT model provided more pronounced risk stratification compared to the RF-CL model, which was also observed for the secondary endpoint (Figure 4). Patients with a high median NCP volume (>41.9 mm^3^) had an increased risk of events regardless of their RF-CL–calculated risk. For example, moderate-risk patients according to the RF-CL model with an NCP volume >41.9 mm^3^ had a 3.8-fold higher risk of MACE compared with moderate-risk patients whose NCP volume was ≤41.9 mm^3^ (Figure 5, Table 5). For the secondary endpoint of death and MI, patients with an NCP volume >41.9 mm^3^ had higher event rates within each RF-CL risk category compared with those in the same category with an NCP volume ≤41.9 mm^3^ (Figure 5).

**Table 5:** Risk Categories According to the RF-CL Model and Corresponding Primary and Secondary Event Rates Stratified by Median NCP Volume

	Primary Endpoint Event Rates % (95% CI)	Secondary Endpoint Event Rates % (95% CI)
Very low risk		
Median NCP volume ≤41.9 mm^3^	2.07% (1.25%-3.42%)	0.82% (0.37%-1.81%)
Median NCP volume >41.9 mm^3^	3.73% (2.12%-6.49%)	2.10% (1.01%-4.35%)
Low risk		
Median NCP volume ≤41.9 mm^3^	1.54% (0.80%-2.94%)	0.52% (0.17%-1.59%)
Median NCP volume >41.9 mm^3^	7.88% (6.02%-10.27%)	2.19% (1.30%-3.67%)
Moderate risk		
Median NCP volume ≤41.9 mm^3^	2.72% (1.14%-6.41%)	2.18% (0.82%-5.72%)
Median NCP volume >41.9 mm^3^	10.24% (7.97%-13.11%)	2.56% (1.52%-4.29%)

Abbreviations as in Tables 3 and 4.

## Discussion

This study represents a multicenter, global registry using AI-guided coronary CT assessment and demonstrated that quantitative atherosclerosis measurements, specifically the burden of noncalcified atherosclerosis and diameter stenosis, were most predictive of future adverse events. Second, AI-QCT significantly enhanced risk stratification for both the primary and secondary endpoints when compared with the RF-CL model, ASCVD score, and traditional risk factors.

CONFIRM2 performed AI-guided plaque quantification of all coronary lesions within the coronary tree from which patient-level measurements were derived. The population consisted of patients with symptoms suggestive of CAD who underwent a clinically indicated CCTA. Most patients (59.5%) were at intermediate likelihood for obstructive CAD, and the event rate was approximately 1% per year. CAD characterization revealed a median TPV of 57 mm^3^, which was equal to a PAV of 2.2%. Most patients had nonobstructive CAD (60%), among which half of the events occurred. The findings of overall noncalcified atheroma burden and stenosis as key predictors for MACE are in line with those of intravascular imaging studies. By IVUS, the atheroma volume is one of the strongest predictors for coronary events.^7^^,^^8^ From OCT, lesions with thin cap fibroatheromas (defined as a lipidic rich plaque with a thin cap) and small lumen area showed the highest rates of subsequent events. Importantly, the plaque volume and LAP quantification by CT have shown high correlation with IVUS and OCT.^11^^,^^12^^,^^14^ ICONIC, a case-control study, demonstrated that the NCP predicted ACS independent from TPV. On the other hand, the so-called “1K plaque” represents a stable phenotype of CAD in which dense calcification exerts a protective effect.^16^^,^^17^ Similarly, the PARADIGM (Progression of AtheRosclerotic PlAque DetermIned by Computed TomoGraphic Angiography IMaging) study highlighted the transformative role of statin therapy in modulating atherosclerotic plaque phenotypes, showing statins to accelerate the calcification of NCPs in patients who underwent serial CCTA imaging.^18^^,^^19^ As such, the process initiated by statin therapy transforms NCPs into a more stable, CP phenotype. A recent retrospective study, however, demonstrated that statin therapy may also modulate low-density CPs (130-199 HU) into denser—and presumably more stable—CP phenotypes.^25^ The importance of plaque burden coupled with the transformative abilities of antiatherosclerotic therapies suggests the need for plaque-based treatment strategy more than risk factor based. The ongoing TRANSFORM trial (Randomized Comparison of Stage-Based Care Versus Risk Factor-Based Care for Prevention of Cardiovascular Events; NCT06112418) evaluates this hypothesis by tailoring antiatherosclerotic therapy by plaque burden and evaluates whether clinical outcomes are improved compared with risk factor–based care. Previously, the SCOT-HEART trial, though not leveraging quantitative CCTA, demonstrated that CCTA-guided management reduced MI incidence by 41% compared to the standard care arm.^26^ This may be a result of more appropriate statin or aspirin usage in patients with the greatest burden of NCP. The importance of diameter stenosis may be attributed to the more advanced and unstable plaque phenotypes of high-grade stenosis, or by recent rapid plaque progression prior to CCTA.^27^ This may have implications for performing revascularization procedures in high-grade coronary stenosis to reduce symptoms or improve prognosis.^28^^,^^29^

The predictive value of total NCP volume across the coronary tree in this CONFIRM2 study highlights the importance of a comprehensive atherosclerotic evaluation. Lesion-specific parameters, such as the number of HRPs or LAP ≥2 mm^3^, did not remain significant after multivariable adjustment, supporting the hypothesis that global plaque burden is more predictive than a focus on HRPs alone, the so-called “vulnerable plaques.” The dynamic nature of atherosclerosis, subclinical plaque ruptures, and widespread use of antiatherosclerotic therapies likely contribute to the limited positive predictive value of vulnerable plaques for future events.^8^ The finding of NCP as the strongest plaque type predictor, as opposed to CP or LAP is unique, since quantitative evaluation of the SCOT-HEART identified LAP to be most prognostic, similar to other studies.^30^, ^31^, ^32^ The discrepant findings with the SCOT-HEART subanalysis may be related to the use of semiquantitative software (Autoplaque) in a selected cohort restricted to patients with visually detectable CAD.^30^ In contrast, the present study did not identify LAP as predictive, which may be explained by different endpoint definitions and the inclusion of all patients irrespective of visually detectable disease. Furthermore, variations in the upper HU threshold for LAP, and whether or not this threshold is adjusted for the lumen contrast attenuation may cause differences in LAP magnitude. In the current study, LAP was characterized by ≥−30 and <30 HU, as it has shown the best accuracy in identifying lipid-rich plaques by near-infrared spectroscopy-IVUS.^11^ In another analysis, Nurmohamed et al within the ISCHEMIA (International Study of Comparative Health Effectiveness With Medical and Invasive Approaches) trial identified TPV as the strongest predictor, but their analysis included only proximal NCP rather than total NCP burden, which likely accounts for the discrepancy with the present findings.^33^ Importantly, TPV, NCP volume, and LAP are strongly correlated,^30^ and while prior studies were limited to a small number of plaque metrics, the current analysis evaluated 24 distinct CT-derived parameters.

AI-driven quantitative CCTA demonstrated significant differences when compared with prior visually scored CT cohorts. For instance, the majority of atherosclerosis in CONFIRM2 was composed of NCP (42 mm^3^) compared to CP (7 mm^3^). Visual assessments, however, often report plaques as predominantly calcified or a mix between calcified and NCP.^34^ In the CONFIRM1 registry, Hadamitzky found that among coronary plaque segments, 17% were noncalcified, 45% were calcified, and 38% had a mixed composition.^34^ The predominance of NCP observed in the current study aligns with findings from histological and IVUS analyses.^35^^,^^36^ These discrepancies between AI-QCT and visual CT readings likely arise from moderate interobserver variability and the underestimation of noncalcified atherosclerosis in qualitative assessments.^37^^,^^38^ AI-QCT classified fewer scans as completely normal because it identifies small volumes of plaque that go undetected with visual evaluation.

Notably, only 7% of the included patients had no plaque, in comparison with 37% and 33% by qualitative CT reads in the SCOT-HEART (Scottish COmputed Tomography of the HEART) and PROMISE (PROspective Multicenter Imaging Study for Evaluation of Chest Pain) trials, respectively, which included patients of comparable age and sex distribution.^26^^,^^39^ In addition, in studies such as the Miami Heart Study and CONFIRM 1 trial, the proportion of patients without CAD has traditionally been around 50%.^40^^,^^41^ Most of these smaller plaques are composed of noncalcified atherosclerosis, which was one of the key predictors in CONFIRM2. The magnitude of the prognostic importance of small volumes of atherosclerosis requires further study with more patients given the low number of events in this subpopulation. A previous serial CCTA study suggests that these plaques are not “image artifact or noise” since they persisted or increased in volume on follow-up CCTA: 87% of all plaques <50 mm^3^ (median 6.2 mm^3^).^42^ Finally, significant differences in plaque volume magnitudes exist between different quantification packages. The median PAV in CONFIRM2 was 2.2%, while this was 39% in patients without events in SCOT-HEART, and 9.1% in another cohort of approximately 11,000 patients; both used different quantification packages.^30^^,^^43^ A recent study by Tzimas et al, including 11,808 patients, aimed to develop nomographic quantitative plaque values using the Artificial Intelligence–Enabled Quantitative Coronary Plaque Analysis tool (HeartFlow).^43^ Reported total, noncalcified, and CP volumes were substantially larger compared with the present study. For example, the median NCP volume was 184 mm^3^ (IQR: 27-486) vs 42.3 mm^3^ (IQR: 13.05-125.55) in the current analysis. This difference may partly be explained by population characteristics, as the cohort studied by Tzimas and colleagues was predominantly from the United States and Canada, potentially reflecting a higher-risk profile compared with the larger proportion of European patients in the current study.^43^ However, CP volumes were also higher in the study by Tzimas et al, which cannot be only explained by vessel wall delineation alone and therefore more likely reflect the higher baseline risk profile of the included patients. Nevertheless, in a subanalysis of the SCOT-HEART trial using Autoplaque (Version 2.5, Cedars-Sinai Medical Center), PAV was substantially larger than in the present study, with a median PAV of 39% among patients without events compared with a median PAV of 2.17% in the overall population of the current analysis.^30^ This is noteworthy, as this involved a European population, yet PAV was higher in SCOT-HEART than in the current cohort. The most likely explanation presumably relates to methodological aspects of plaque quantification, particularly the delineation of the vessel wall, which may have contributed to the higher PAV observed in SCOT-HEART. Inclusion of the vessel wall in seemingly normal coronary segments can markedly increase the estimated NCP volume. This methodological difference may also explain the lower plaque volumes observed in CONFIRM2, where only plaque from individual coronary lesions was quantified.

### Study limitations

This study is not without limitations. First, medical treatment may have been initiated or adjusted based on coronary CT findings, potentially influencing the results. Such interventions could attenuate the prognostic impact of the atherosclerosis findings, likely resulting in conservative effect size estimates. Additionally, all coronary CT scans were analyzed using an AI-driven model without a comparison to the performance of visual coronary CT assessments. Studies directly comparing AI-QCT with CAD-RADS 2.0 are warranted, as such a visual benchmark would have further strengthened the findings of the present analysis. Furthermore, the absence of the coronary calcium score for all patients precluded a direct comparison, although a strong correlation exists between the Agatston score and CP volume on coronary CTA (R = 0.76).^44^

## Conclusions

This first multicenter global registry with AI-guided quantitative CT identified NCP burden and increment in stenosis severity as the most powerful predictors of MACE outperforming the RF-CL model and the ASCVD risk score. This demonstrates the interplay between traditional and novel measures of the severity of CAD. Standardized and rapid quantitative assessment of CAD may improve clinical implementation of multidimensional assessment of CAD as a cornerstone for risk assessment.

## Funding support and author disclosures

The study is sponsored by Cleerly, Inc. Dr van Rosendael is a member of the Cleerly Scientific Advisory Board. Dr Pontone has received honorarium as speaker/consultant and/or institutional research grant from GE Healthcare, Bracco, Medtronic, Novartis. Dr Buechel reports receiving speaking honoraria from GE Healthcare, Pfizer, Gilead, and IBA. Dr Gräni received funding from the Swiss National Science foundation, InnoSuisse, CAIM foundation, GAMBIT foundation, Novartis foundation for biomedical research, outside of the submitted work. Dr Choi is a consultant for Siemens, holds equity in Cleerly, and receives grant support from the George Washington Heart and Vascular Institute. Dr Rochitte reports receiving speaking honoraria for Pfizer, Edwards, GE, and Manole. Dr Knaapen received research grants from Cleerly Inc, and HeartFlow. Dr Khalique is a consultant for Edwards, Croivalve, Restore Medical, holds equity in Triflo, and has received honoraria for educational programs from Heartflow. Dr Marques is a consultant for Cleerly, Inc. Dr Aquino is an employee, Cleerly, Inc. Dr Danad is a member of the Cleerly Scientific Advisory Board and received a research grant from Cleerly Inc. All other authors have reported that they have no relationships relevant to the contents of this paper to disclose.
