# Supplementary material for: AI-Driven Quantitative Coronary CT Angiography in Suspected Coronary Artery Disease: Multicenter CONFIRM2 Registry
Source: JACC Adv. 2026 Mar 25;5(3):102618. doi: 10.1016/j.jacadv.2026.102618 (PMC13352004; doi:10.1016/j.jacadv.2026.102618)
Supplement: Supplemental Material [file mmc1.docx]

**APPENDIX**

| **APPENDIX Table 1. Definitions of AI-QCT variables** | |
| --- | --- |
| **Quantitative CT feature** | **Definition** |
| Diameter stenosis, per 10% | Maximal patient-level diameter stenosis |
| Diameter Area, per 10% | Maximal patient-level area stenosis |
| Number of moderate stenosis (50-69%) (0,1,2,>=3) | Number of stenosis 50-70% within a patient |
| Number of severe stenosis (>=70%) (0,1,>=2) | Number of stenosis >=70% within a patient |
| Total plaque volume, per 50 mm3 | Total plaque volume summed from all coronary lesions within a patient |
| Calcified plaque volume, per 50 mm3 | Total calcified plaque volume (>350 HU) summed from all coronary lesions within a patient |
| Non-calcified plaque volume, per 50 mm3 | Total non-calcified plaque volume (between -30 and +350 summed from all lesions segments within a patient |
| Low-density noncalcified plaque volume, per 1 mm3 | Total low-density plaque volume (-30 to <30 HU) summed from all coronary lesions within a patient |
| Percent atheroma volume (PAV), per 1% | Plaque volume / vessel volume *100%, derived from all coronary segments within a patient |
| PAV calcified plaque, per 1% | Calcified plaque volume / vessel volume *100%, derived from all coronary segments within a patient |
| PAV non-calcified plaque, per 1% | Non-calcified plaque volume / vessel volume *100%, derived from all coronary segments within a patient |
| PAV Low-attenuation plaque, per 1% | Low-attenuation plaque volume / vessel volume *100%, derived from all coronary segments within a patient |
| # of high-risk plaques (0,1,2,3,>3) | Number of lesions with both LAP ≥2mm^3^ and a remodeling index >1.1 within a patient |
| Plaque diffuseness | Plaque volume / vessel length within a patient |
| Total Vessel Length | Summed length of the coronary segments within a patient |
| Left main with moderate stenosis | Left main with 50-69% stenosis |
| Left main with severe stenosis | Left main with >/=70% stenosis |
| Proximal LAD with moderate stenosis | Proximal LAD with 50-69% stenosis |
| Proximal LAD with severe stenosis | Proximal LAD with >/=70% stenosis |
| Number of CTO (100% stenosis) | Number of CTO lesions within a patient |
| Lumen volume | Total lumen volume summed from all coronary segments within a patient |
| Vessel volume | Total vessel volume summed from all coronary segments within a patient |
| Lumen volume / vessel length (average lumen area; mm3/mm2) | - |
| Minimal lumen diameter | Minimal lumen diameter within a patient |

LAD, left anterior descending artery; CTO, chronic total occlusion

| **APPENDIX Table 2. Baseline Medication usage** |  |
| --- | --- |
| **Variables** | **All  Patients   N =  3551** |
| Statin |  |
| High intensity (>=40 mg atorvastatin or >=20 mg rosuvastatin) | 248(7.0%) |
| Low to moderate intensity (all others) | 989(27.9%) |
| No | 716(20.2%) |
| Yes (unspecified drug/dosage) | 239(6.7%) |
| Anti-platelet medication |  |
| No | 2932(82.6%) |
| Yes | 169(4.8%) |
| Ace inhibitor or ARB |  |
| No | 1377(38.8%) |
| Yes | 1154(32.5%) |
| Calcium channel blocker |  |
| No | 1965(55.3%) |
| Yes | 568(16.0%) |
| Beta blocker |  |
| No | 1286(36.2%) |
| Yes | 1258(35.4%) |
| Nitrates |  |
| Long-acting | 44(1.2%) |
| No | 2042(57.5%) |
| Short-acting (sublingual / spray) | 61(1.7%) |
| Any antidiabetic medication |  |
| No | 2860(80.5%) |
| Yes | 243(6.8%) |

| **APPENDIX Table 3. Univariable quantitative CT candidates for the optimal multivariable AI-QCT model** | | |
| --- | --- | --- |
| **Quantitative CT feature** | **Hazard Ratio (95% CI)** | **HR  P-value** |
| Diameter stenosis, per 10% | 1.32 (1.26,1.38) | <.0001 |
| Diameter Area, per 10% | 1.33 (1.26,1.39) | <.0001 |
| Number of moderate stenosis (50-69%) (0,1,2,>=3) | 2.01 (1.72,2.34) | <.0001 |
| Number of severe stenosis (>=70%) (0,1,>=2) | 2.69 (2.07,3.49) | <.0001 |
| Total plaque volume, per 50 mm3 | 1.07 (1.06,1.09) | <.0001 |
| Calcified plaque volume, per 50 mm3 | 1.11 (1.08,1.13) | <.0001 |
| Non-calcified plaque volume, per 50 mm3 | 1.14 (1.12,1.17) | <.0001 |
| Low-density noncalcified plaque volume, per 1 mm3 | 1.01 (1.00,1.02) | 0.0235 |
| Percent atheroma volume (PAV), per 1% | 1.07 (1.06,1.08) | <.0001 |
| PAV calcified plaque, per 1% | 1.08 (1.06,1.10) | <.0001 |
| PAV non-calcified plaque, per 1% | 1.15 (1.12,1.17) | <.0001 |
| PAV Low-attenuation plaque, per 1% | 1.75 (1.18,2.60) | 0.0055 |
| # of high risk plaques (0,1,2,3,>3) | 1.41 (1.18,1.69) | 0.0002 |
| Plaque diffuseness | 2.47 (2.09,2.92) | <.0001 |
| Total Vessel Length | 1.00 (1.00,1.00) | 0.7129 |
| Left main with moderate stenosis | 6.04 (1.93,18.93) | 0.0020 |
| Left main with severe stenosis | No events | – |
| Proximal LAD with moderate stenosis | 2.42 (1.69,3.46) | <.0001 |
| Proximal LAD with severe stenosis | 4.74 (2.91,7.74) | <.0001 |
| Number of CTO (100% stenosis) | 1.67 (1.31,2.13) | <.0001 |
| Lumen volume | 1.00 (1.00,1.00) | 0.5271 |
| Vessel volume | 1.00 (1.00,1.00) | 0.0007 |
| Lumen volume / vessel length (average lumen area; mm3/mm2) | 1.04 (0.92,1.18) | 0.4943 |
| Minimal lumen diameter | 0.46 (0.38,0.56) | <.0001 |

For identifying the Optimal quantitative CT Model, each of the following 24 CT parameters were assessed univariately to see the individual risk. The CT parameters were then assessed in a logistic regression model to exclude collinear variables defined as a variance inflation factor>5. Once collinear variables were excluded, a Multivariate Cox Regression model was fit and backwards selection at P<0.05 was used to identify the best predictors. Non-Calcified plaque and % Diameter stenosis were the variables that remained in the Optimal AI-QCT Model.

| **APPENDIX Table 4A: Primary Endpoint by Plaque Tertiles** | | | | | |
| --- | --- | --- | --- | --- | --- |
|  | **Event Rate** | **Hazard Ratio**  **(95% CI)** | **Hazard Ratio P-value** | **Hazard Ratio Per Tertile Increase**  **(95% CI)** | **HR P-value** |
| TPV |  |  |  |  |  |
| 0-26 | 1.7% (20/1185) | Ref. |  | 2.72 (2.16,3.43) | <.0001 |
| >26-126 | 2.8% (33/1182) | 1.67 (0.96,2.92) | 0.0689 |  |  |
| >126 | 9.6% (114/1184) | 6.01 (3.74,9.67) | <.0001 |  |  |
| NCP |  |  |  |  |  |
| 0-20.5 | 1.7% (20/1186) | Ref. |  | 2.80 (2.22,3.54) | <.0001 |
| >20.5-82.6 | 2.6% (31/1181) | 1.57 (0.90,2.76) | 0.1147 |  |  |
| >82.6 | 9.8% (116/1184) | 6.13 (3.81,9.85) | <.0001 |  |  |
| CP |  |  |  |  |  |
| 0-0.5 | 1.8% (21/1190) | Ref. |  | 2.22 (1.79,2.75) | <.0001 |
| >0.5-28.1 | 3.9% (46/1177) | 2.25 (1.34,3.78) | 0.0020 |  |  |
| >28.1 | 8.5% (100/1184) | 4.97 (3.10,7.95) | <.0001 |  |  |

| **APPENDIX Table 4B: Secondary Endpoint by Plaque Tertiles** | | | | | |
| --- | --- | --- | --- | --- | --- |
|  | **Event Rate** | **Hazard Ratio**  **(95% CI)** | **Hazard Ratio P-value** | **Hazard Ratio Per Tertile Increase**  **(95% CI)** | **HR P-value** |
| TPV |  |  |  |  |  |
| 0-26 | 0.9% (11/1185) | Ref. |  | 2.17 (1.51,3.12) | <.0001 |
| >26-126 | 0.8% (9/1182) | 0.83 (0.34,1.99) | 0.6693 |  |  |
| >126 | 3.2% (38/1184) | 3.53 (1.80,6.91) | 0.0002 |  |  |
| NCP |  |  |  |  |  |
| 0-20.5 | 0.9% (11/1186) | Ref. |  | 2.33 (1.61,3.38) | <.0001 |
| >20.5-82.6 | 0.6% (7/1181) | 0.64 (0.25,1.65) | 0.3577 |  |  |
| >82.6 | 3.4% (40/1184) | 3.72 (1.91,7.26) | 0.0001 |  |  |
| CP |  |  |  |  |  |
| 0-0.5 | 0.7% (8/1190) | Ref. |  | 2.02 (1.42,2.88) | <.0001 |
| >0.5-28.1 | 1.4% (17/1177) | 2.17 (0.94,5.03) | 0.0705 |  |  |
| >28.1 | 2.8% (33/1184) | 4.21 (1.94,9.11) | 0.0003 |  |  |

TPV, total plaque volume; NCP, noncalcified plaque (volume); CP, calcified plaque (volume); CI, confidence interval.

| **APPENDIX Table 5. Multivariable modeling for the prediction of the secondary outcome myocardial infarction and death** | | | |
| --- | --- | --- | --- |
| **Variables** | **Multivariable HR (95%CI)** | **AUC** | **P-value Vs. RF-CL** |
| **RF-CL*** |  | 0.574 (0.493, 0.654) | p < .0001 (p-value testing the hypothesis that c=0.5) |
| Very Low  Low  Moderate | Ref.  1.119 (0.544, 2.305)  1.997 (0.978, 4.076) | -- | -- |
|  |  |  |  |
| **ASCVD*** |  | 0.604 (0.526, 0.682) | 0.3333 (vs. RF-CL model) |
| ASCVD risk score  0-5%  5-7.5%  7.5-20%  >20% | Ref.  1.926 (0.431, 8.604)  4.206 (1.424, 12.428)  2.892 (0.871, 9.605) | -- | -- |
|  |  |  |  |
| **Optimal AI-QCT model** |  | 0.736 (0.672, 0.800) | 0.0013 (vs. RF-CL model) |
| Lumen diameter stenosis, per 10% | 1.177 (1.066,1.299) | -- | -- |
| Noncalcified plaque volume, per 50 mm^3^ | 1.097 (1.042, 1.154) | -- | -- |
|  |  |  |  |
| **Risk factors + Age + Sex + Optimal AI-QCT model** |  | 0.753 (0.688, 0.818) | 0.0001 (vs. RF-CL model) |
| Diabetes | 1.077 (0.522, 2.219) | -- | -- |
| Hypertension | 1.087 (0.590, 2.002) | -- | -- |
| Smoking | 2.426 (1.293, 4.554) | -- | -- |
| Dyslipidemia | 0.807 (0.451, 1.442) | -- | -- |
| Age | 1.011 (0.984, 1.039) | -- | -- |
| Sex (Male vs. Female) | 1.201 (0.632, 2.283) | -- | -- |
| Lumen diameter stenosis, per 10% | 1.131 (1.009, 1.269) | -- | -- |
| Noncalcified plaque volume, per 50 mm^3^ | 1.103 (1.041, 1.169) | -- | -- |
|  |  |  |  |
| **ASCVD + Optimal AI-QCT model** |  | 0.721 (0.627, 0.814) | 0.0070 (vs. RF-CL model) |
| ASCVD risk score  0-5%  5-7.5%  7.5-20%  >20% | Ref.  1.614 (0.359, 7.267)  2.723 (0.879, 8.436)  1.615 (0.456, 5.724) | -- | -- |
| Lumen diameter stenosis, per 10% | 1.128 (0.980, 1.298) | -- | -- |
| Noncalcified plaque volume, per 50 mm^3^ | 1.076 (1.005, 1.153) | -- | -- |
|  |  |  |  |
| **RF-CL + Optimal AI-QCT model** |  | 0.733 (0.661, 0.805) | 0.0020 (vs. RF-CL model) |
| RF-CL risk score  Very Low  Low  Moderate | Ref.  0.849 (0.407, 1.775)  0.954 (0.429, 2.123) | -- | -- |
| Lumen diameter stenosis, per 10% | 1.143 (1.019, 1.280) | -- | -- |
| Noncalcified plaque volume, per 50 mm^3^ | 1.110 (1.049, 1.174) | -- | -- |
|  |  |  |  |
| **RF-CL + Optimal AI-QCT model + Statins** |  | 0.772 (0.690, 0.854) | 0.0103 (vs. RF-CL model) |
| RF-CL risk score  Very Low  Low  Moderate | Ref.  0.816 (0.262, 2.545)  1.031 (0.320, 3.324) | -- | -- |
| Lumen diameter stenosis, per 10% | 1.241 (1.060, 1.453) | -- | -- |
| Noncalcified plaque volume, per 50 mm^3^ | 1.059 (0.960, 1.170) | -- | -- |
| Statins* | 0.874 (0.339, 2.252) | -- | -- |
| *ASCVD score was available in 1,309 patients, Statins were available in 2,192 patients and RF-CL was available in 3052 patients  RF-CL; risk factor-weighted clinical likelihood model; ASCVD, Atherosclerotic Cardiovascular Disease risk score; AI-QCT; Artificial Intelligence–Guided Quantitative CCTA; AUC, area under the curve. | | | |

| **APPENDIX Table 7. Subcohort: Amongst Females (N=1,759), primary endpoint** | | | |
| --- | --- | --- | --- |
| **Model** | | **AUC (95% CI)** | **P-value**  **Vs. RF-CL** |
|  | |  |  |
| *Clinical scores* | |  |  |
| RF-CL Risk Score | | 0.569 (0.498, 0.640) | -- |
| ASCVD Risk Score | | 0.608 (0.521, 0.695) | 0.2548 |
|  | |  |  |
| *CCTA scores* | |  |  |
| Optimal AI-QCT Model: stenosis diameter and noncalcified plaque volume | | 0.755 (0.691, 0.820) | <.0001 |
| Abbreviations as in Appendix Table 6. |  | |  |

| **APPENDIX Table 6. Subcohort: Amongst Males (N=1,792), primary endpoint** | | |
| --- | --- | --- |
| **Model** | **AUC (95% CI)** | **P-value**  **Vs. RF-CL** |
|  |  |  |
| *Clinical scores* |  |  |
| RF-CL Risk Score | 0.602 (0.552, 0.653) | -- |
| ASCVD Risk Score | 0.625 (0.560, 0.691) | 0.6435 |
|  |  |  |
| *CCTA scores* |  |  |
| Optimal AI-QCT Model: stenosis diameter and noncalcified plaque volume | 0.733 (0.688, 0.777) | <.0001 |
|  |  |  |
| Abbreviations: CCTA, coronary computed tomography angiography. Other abbreviations as in Appendix Table 4. | | |

| **APPENDIX Table 8. Subcohort: Amongst Age <55 years (N=1,248), primary endpoint** | | |
| --- | --- | --- |
| **Model** | **AUC (95% CI)** | **P-value**  **Vs. RF-CL Risk Score** |
|  |  |  |
| *Clinical scores* |  |  |
| RF-CL Risk Score | 0.595 (0.505,0.685) | -- |
| ASCVD Risk Score | 0.646 (0.528, 0.764) | 0.3565 |
|  |  |  |
| *CCTA scores* |  |  |
| Optimal AI-QCT Model:  stenosis diameter and noncalcified plaque volume | 0.764 (0.690,0.838) | <.0001 |
|  |  |  |
| Abbreviations as in Appendix Table 6. | | |

| **APPENDIX Table 9. Subcohort: Amongst Age >55 years (N=2,303), primary endpoint** | | |
| --- | --- | --- |
| **Model** | **AUC (95% CI)** | **P-value**  **Vs. RF-CL Risk Score** |
|  |  |  |
| *Clinical scores* |  |  |
| RF-CL Risk Score | 0.613 (0.565, 0.661) | -- |
| ASCVD Risk Score | 0.581 (0.519, 0.642) | 0.6266 |
|  |  |  |
| *CCTA scores* |  |  |
| Optimal AI-QCT Model: stenosis diameter and noncalcified plaque volume | 0.737 (0.694, 0.780) | <.0001 |
|  |  |  |
| Abbreviations as in Appendix Table 6. | | |

| **APPENDIX Table 10. Subcohort: Amongst patients without obstructive CAD (N=3,034), primary endpoint** | | |
| --- | --- | --- |
| **Model** | **AUC (95% CI)** | **P-value**  **Vs. RF-CL Risk Score** |
|  |  |  |
| *Clinical scores* |  |  |
| RF-CL Risk Score | 0.615 (0.554, 0.676) | -- |
| ASCVD Risk Score | 0.594 (0.521, 0.667) | 0.8828 |
|  |  |  |
| *CCTA scores* |  |  |
| Optimal AI-QCT Model: stenosis diameter and noncalcified plaque volume | 0.698 (0.647, 0.747) | 0.0014 |
|  |  |  |
| Abbreviations as in Appendix Table 6. | | |

| **APPENDIX Table 11.**  **Comparison of Predictive Value Using Harrell’s C and AUC** | | |
| --- | --- | --- |
| **Variables** | **Harrell’s** | **AUC** |
| RF-CL | 0.622 | 0.625 |
| ASCVD | 0.629 | 0.632 |
| AI-QCT model | 0.752 | 0.755 |
| Risk factors + Age + Sex + Optimal AI-QCT model | 0.770 | 0.774 |
| ASCVD + Optimal AI-QCT model | 0.747 | 0.747 |
| RF-CL + Optimal AI-QCT model | 0.756 | 0.762 |
| RF-CL + Optimal AI-QCT model + Statins | 0.754 | 0.758 |

Abbreviations as in Appendix Table 5.

**Appendix Figure 1. Histogram of non-calcified plaque.**


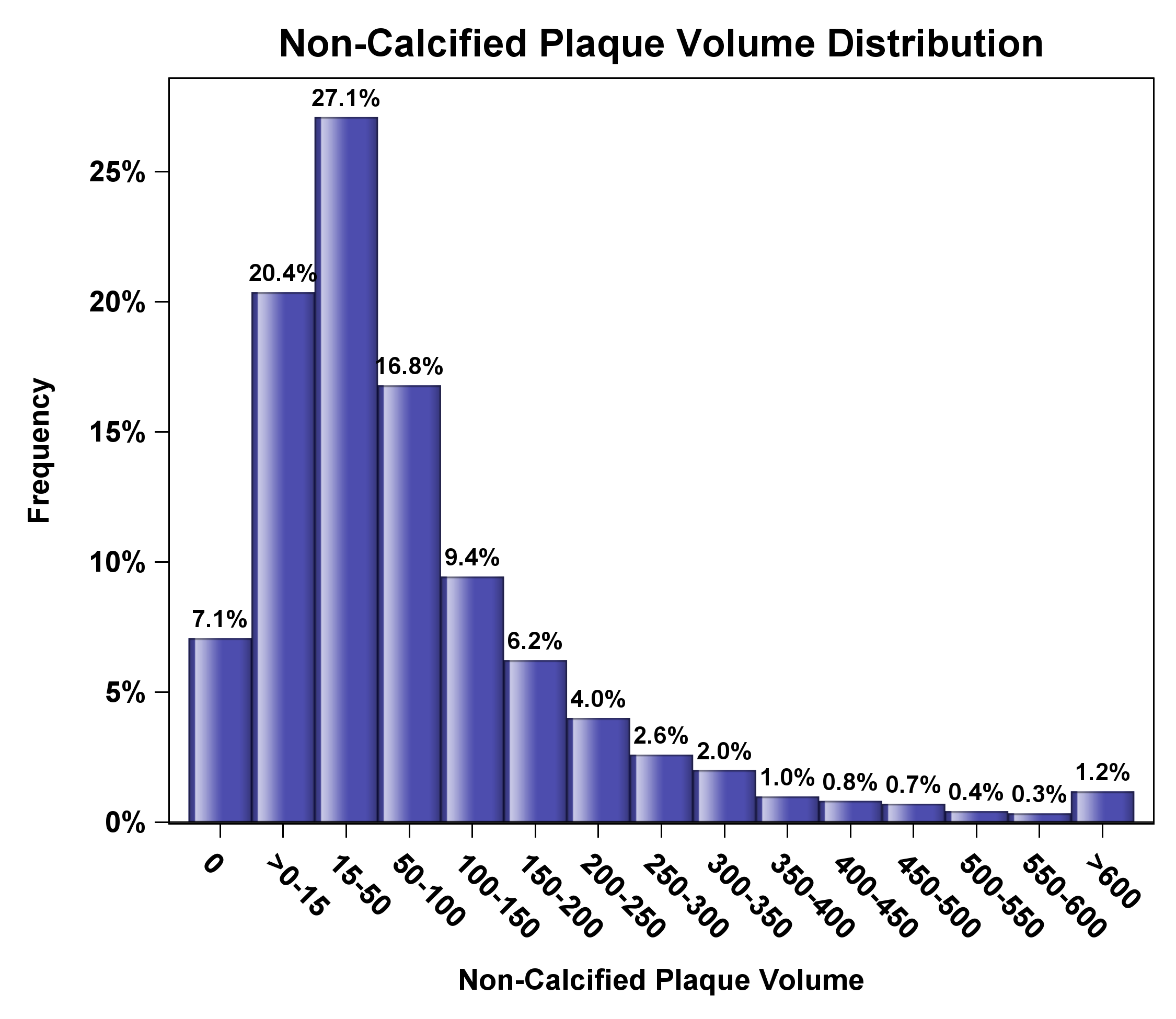


**Appendix Figure 2. Predicted probability of events for the primary endpoint by diameter stenosis (A), TPV (B) and NCP volume (C).**

**Appendix Figure 2A**


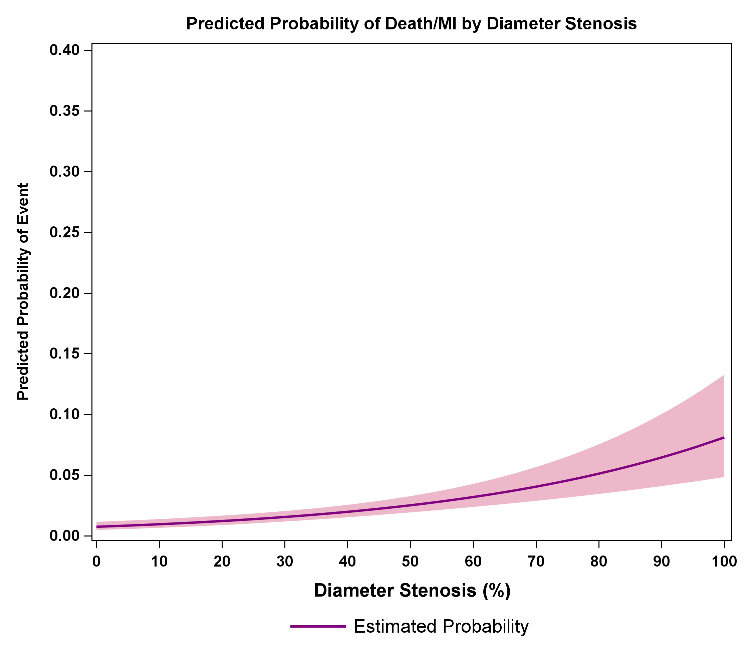


**Appendix Figure 2B**


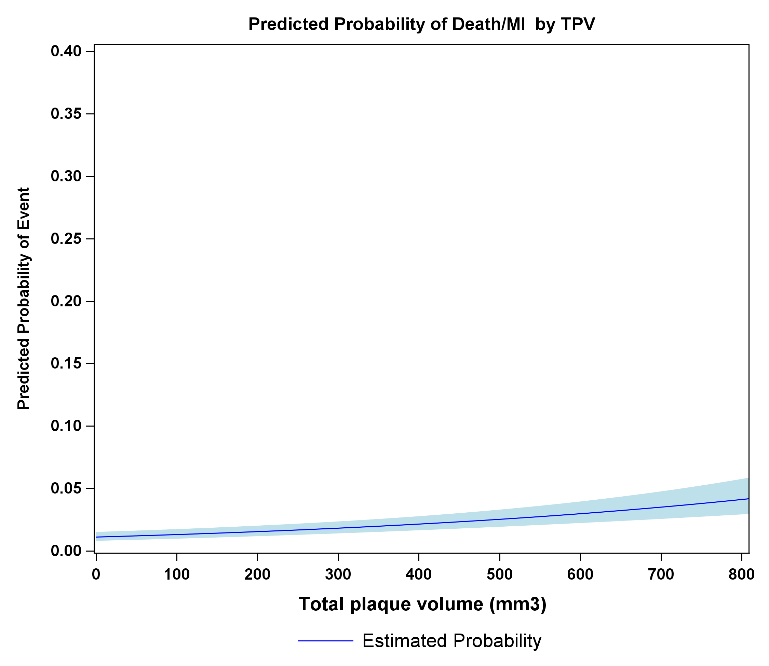


**Appendix Figure 2C**


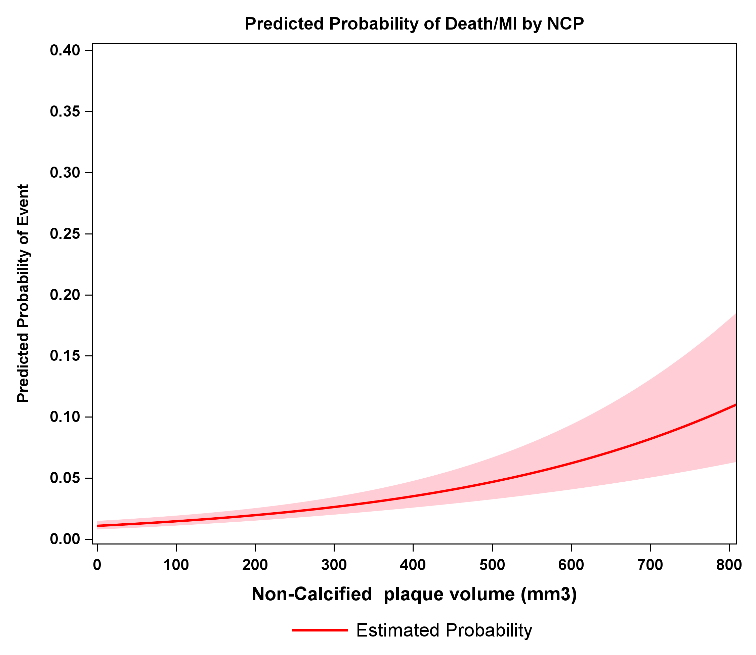


TPV, total plaque volume; NCP, noncalcified plaque, MI, myocardial infarction
